# Topohistology of dendritic cells and macrophages in the distal and proximal nodes along the lymph flow from the lung

**DOI:** 10.1111/joa.14251

**Published:** 2025-03-28

**Authors:** Masaya Aoki, Go Kamimura, Aya Harada‐Takeda, Toshiyuki Nagata, Gen Murakami, Kazuhiro Ueda

**Affiliations:** ^1^ Department of General Thoracic Surgery, Graduate School of Medical and Dental Sciences Kagoshima University Kagoshima Japan; ^2^ Department of Anatomy Tokyo Dental College Tokyo Japan

**Keywords:** anti‐cancer immunity, CD169‐positive macrophages, dendritic cells, lung regional node, morphometry, preconditioning along lymph flow, tumor size

## Abstract

Nodal dendritic cells and CD169‐positive macrophages cross‐present cancer antigens earlier in the proximal nodes than in the distal nodes along the lymph flow from cancer. We examined topohistological differences between the proximal and distal nodes before the formation of metastasis. Immunohistochemical and morphometric analyses were performed to examine DC‐SIGN‐, CD68‐, and CD169‐positive cells in the subcarinal node (proximal) and paratracheal nodes (distal nodes) from 16 patients with lower‐lobe lung cancer without metastasis (adenocarcinoma, 11; squamous, 5). Nodes at the same sites from 10 patients with upper‐lobe cancer were used as controls. In all nodes, the medullary sinus was filled with CD68‐positive and CD169‐negative macrophages, most of which showed anthracosis. The proximal node carried a significantly smaller overlap between clusters of DC‐SIGN‐positive cells and CD169‐positive cells relative to the distal node in lower‐lobe cancer patients (*p* = 0.015). Irrespective of the cancer pathology, the tumor size was significantly correlated with the longer subcapsular clusters containing either DC‐SIGN‐positive cells or CD169‐positive cells (*p* = 0.003, 0.043). A significantly small overlap between these clusters as well as the missing paracortical sinuses was evident in the negative control node outside the lymph flow (*p* = 0.006). Since DC‐SIGN‐positive cells and CD169‐positive cells, especially composite cells in the overlapped cluster, are likely to be derived from monocytes, larger tumors appeared to accelerate the migration into the subcapsular sinus. In contrast to the suggested active status of the distal node, the proximal node appeared to have already been suppressed. This downregulation reached the level in the negative control node.

## Graphical abstract

Proximal node (subcarinal node) from a patient with lower‐lobe lung cancer. The complementary distribution was seen between CD68‐positive macrophages and CD169‐positive cells, and the latter tended to exist with DCsign‐positive cells. The island‐like appearance of the cortex was seen in the nodes. Anthracotic macrophages with carbon particles were observed in the medullary sinus. Overall, there was not a clear difference in general histology between the proximal and distal nodes.

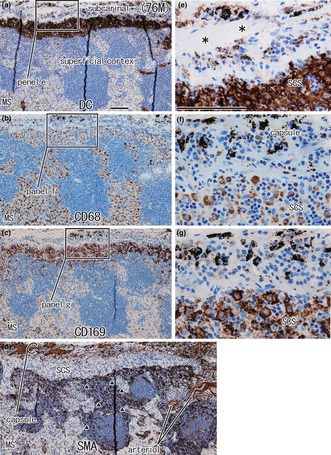

## 1 INTRODUCTION

Nodal dendritic cells (DCs) and macrophages are considered key professional antigen‐presenting cells in cancer immunity (Kvedaraite & Ginhoux, 2022). In this context, this definition did not include follicular DCs. However, there is substantial evidence that macrophages are immunosuppressive and often preconditioned to promote nodal metastasis (Blayer et al., 2022; Commerford et al., 2018; Du et al., 2021; Gunnarsdottir et al., 2023; Heeren et al., 2021; Ji et al., 2024; Mehta et al., 2021; van Pul et al., 2019; Virgilio et al., 2022; Wei et al., 2021). A limited exception of cancer‐induced tolerance is seen in CD169 (Siglec1 or sialoadhesin)‐positive macrophages (Gunnarsdottir et al., 2023) that cross‐present cancer antigens (Grabowska et al., 2018; Reis‐Sobreiro et al., 2021). CD169‐positive macrophages, a unique subset that differs from M1 and M2 populations (Liu et al., 2021), enhance the cross‐priming of T lymphocytes by DCs with the localized production of type I interferons in the cancer regional node, resulting in a better prognosis (Fujiwara et al., 2024; Kumamoto et al., 2021; Rakaee et al., 2019).

Based on immunohistochemical staining of DC‐SIGN (hereafter DCsign: DC‐specific ICAM‐3‐grabbing nonintegrin; CD209)‐positive cells, our group recently demonstrated morphological differences between the sentinel and non‐sentinel nodes of early gastric cancer patients without metastasis (Sonoda et al., 2025). DCsign‐positive cells and CD169‐positive cells usually coexist in the subcapsular and paracortical sinuses (PCSs), and the overlapping area of clusters is significantly smaller in sentinel nodes than in non‐sentinel nodes, possibly due to suppression by cancer. Increased suppression has been reported in sentinel nodes relative to non‐sentinel nodes (Botella‐Estrada et al., 2005; Cochran et al., 2001; Matsuura et al., 2006; Otto et al., 2014; van Pul et al., 2020), but non‐sentinel nodes are not usually located on the distal or downstream side of the sentinel node; rather, they are usually “outside” of the lymphatic flow. Therefore, in the present study, we aimed to compare the proximal node with the distal node to identify topohistological differences in candidate DCs and macrophages such as dominancy in the paracortical or medullary sinus (MS) as well as the size of the occupied area.

In lung regional nodes, we demonstrated the site‐specific distribution of nodal DCsign‐positive cells and CD68‐positive macrophages (Aoki et al., 2023). In short, these cell populations did not overlap in distribution: DCsign‐positive cells in the subcapsular, intermediate, and PCSs, in contrast to the macrophages in the MS and cortex. Sinus endothelia also express DCsign (Engering et al., 2004; Lai et al., 2006; Jin et al., 2022; Park et al., 2014; Aoki et al., 2023; Sonoda et al., 2025). In the present study, we continued to note the topohistological relation between nodal macrophages and candidate DCs. According to anatomical studies using cadavers, lymph flow from the lower lung lobe reaches the ipsilateral paratracheal node via the subcarinal node, while the upper lobe drains the lymph directly to the paratracheal node (Jossifow, 1930; Murakami et al., 1990; Riquet, 1993; Riquet et al., 1989, 1991; Yano, 1993). The result of these cadaveric studies was strongly supported by large retrospective analyses of cancer metastasis (Aokage et al., 2010; Asamura et al., 1999; Little et al., 1999; Naruke et al., 1978; Okada et al., 1998). Accordingly, we chose the subcarinal node (proximal node) and lower paratracheal node (distal node) in patients with early lower‐lobe cancer without metastasis. Nodes at the same sites from 10 patients with upper‐lobe cancer were used as controls.

## 2 MATERIALS AND METHODS

### 2.1 Patients and nodal specimens

Eighty thoracic nodes (35 subcarinal nodes and 45 paratracheal nodes) were examined morphologically and immunohistochemically (Table 1). Nodes were surgically obtained from 16 non‐small cell lung cancer patients without metastasis (male, *n* = 9; female, *n* = 7; age: 56–78 years). Eleven of these 16 patients were smokers. Sixteen patients underwent curative pulmonary resection with lymph node dissection at the Kagoshima University Hospital between April 2010 and March 2021. After routine curative resection and nodal dissection (lobectomy, *n* = 15; bi‐lobectomy, *n* = 1), all patients survived for >5 years without any recurrence. The final pathological examination revealed 5 patients with stage IA, 8 patients with stage IB, 2 patients with stage IIB, and 1 patient with stage IIIA lung cancer. The cancers were histopathologically classified as adenocarcinoma (*n* = 11) or squamous cell carcinoma (*n* = 5), according to the seventh edition of the TNM classification (Goldstraw et al., 2007).

**TABLE 1:** Lymph node examined.

Site of nodes	Numbers of node	Nodal area mean mm^2^	DCsign cluster/node area mean %^a^	CD68/node area mean %^b^	CD169/node area mean %^c^
Lower‐lobe cancer patients					
Proximal nodes	22	24.7	14.3	38.0	13.7
Distal nodes	25	22.1	17.0	40.9	17.9
Upper‐lobe cancer patients					
Candidate proximal nodes	20	29.2	13.6	49.4	10.2
Outsider nodes	13	35.9	13.3	53.2	8.2

^a^ A proportion (%) of DCsign‐positive cell cluster area in the nodal area.

^b^ A proportion (%) of CD68‐positive macrophage cluster area in the nodal area.

^c^ A proportion (%) of CD169‐positive cell cluster area in the nodal area.

The removed nodes were fixed in 10% w/w neutral formalin solution for 7 days, followed by a routine procedure for paraffin‐embedded histology. From one node, we prepared 5–6 serial sections, including the maximum sectional area in the node. One section was stained with hematoxylin and eosin (HE), while the other was used for immunohistochemistry (see subsection below). Because the paratracheal or subcarinal site of 1 patient contained multiple nodes (a maximum of 12 nodes), we chose 1–3 nodes for morphometry. Nodes at the same sites from 10 patients (male, *n* = 5; female, *n* = 5; age, 50–74 years) with “upper lobe” cancer were used as controls. In patients with upper‐lobe lesions, the paratracheal node corresponds to the candidate proximal node (positive control), while the subcarinal node is most likely to be an “outsider node” (negative control) that is located outside of the lymph flow from the primary lesion. The use of specimens was approved by the Ethics Committee of Kagoshima University Graduate School of Medicine and Dentistry (No. 210198epi).

### 2.2 Immunohistochemistry

The primary antibodies, together with their dilutions and antigen retrieval procedures, are listed in Table 2. Briefly, we used (1) an antibody against DCsign (also known as CD209; for more detail, see second paragraph of Section 1) as a DC marker, (2) a pan‐macrophage marker CD68, (3) another marker for the macrophage subpopulation CD169 (for more detail, see third paragraph of Section 1), and (4) an antibody for alpha smooth muscle actin (SMA) in the sinus endothelium. The paracortical lymph sinus endothelium expresses both SMA and DCsign (Aoki et al., 2023). After incubation with primary antibodies, the sections were incubated for 30 min with horseradish peroxidase (HRP)‐conjugated secondary antibodies (Histofine Simple Stain Max‐PO; Nichirei, Tokyo, Japan) diluted 1:1000. Immunoreactive proteins were detected by incubation with diaminobenzidine for 3–5 min (Histofine Simple Stain DAB; Nichire). Each sample was counterstained with hematoxylin, and a negative control without the primary antibody was used for all specimens. Treated sections were counterstained with hematoxylin, dehydrated in ethanol, and cleared in xylene. All histological images were captured using a Nikon Eclipse 80i microscope.

**TABLE 2:** Primary monoclonal antibodies, their dilution, and specific treatment.

Legend	Ig types	Sources	Final dilution	Antigen retrieval
DC‐sign	Mouse	Santa Cruz sc‐65,740 (TX, USA)	1:200	Dako PT Link pH high
CD169	Rabbit	Abcam ab183356 (Cambridge, UK)	1:100	Citrate buffer
CD68	Mouse	Dako N0814 (Grostrup, Denmark)	1:200	Trypsin
SMA	Mouse	Dako M0851 (Grostrup, Denmark)	1:800	Trypsin

Abbreviation: SMA, α smooth muscle actin.

Yamada et al. (2023) reported DCsign‐ CD169‐double positive cells in human nodes, but we considered them a minor population according to Aoki et al. (2023). Because the current study started from a topohistological interest (see Section 1), the numbers, density, and distribution of the suggested double‐positive cells were out of focus.

### 2.3 Morphometric analysis of clusters of candidate DCs and macrophages

Using stained sections corresponding to the maximum cross‐sectional area of the node, we measured four parameters of the area and four parameters of length: (1) the entire sectional area of the nodes, (2) the proportional area of DCsign‐positive cell clusters, (3) the proportional area of CD68‐positive macrophage clusters, (4) the proportional area of CD169‐positive cell clusters, (5) the entire length of the node circumference, and (6–8) a circumferential lengths of the subcapsular sinus (SCS) containing each of those clusters.

First, we prepared three photos of histology (x28 magnification; 1 mm in the histology corresponded to 28 mm on the photo) each of which showed either DCsign‐positive cell clusters, CD68‐positive macrophage clusters or CD169‐positive cell clusters in the entire section of a node. Next, each photo was covered with a tracing paper and we manually traced the island‐like clusters as well as the SCS by black ink. The tracing paper was scanned using a high‐grade flat scanner with translucent illumination (Epson scanner GTX970). The digital data was moved to Adobe Photoshop. We processed the data using ImageJ (version 1.53; U.S. National Institutes of Health) to provide the cluster area (mm^2^). During the procession, simultaneously, we obtained the entire circumferential length of the node as well as a specific part of the circumference (= a subcapsular length of the sinus containing clusters of each type of cells examined). Differences between values were statistically analyzed using Student's *t*‐test.

## 3 RESULTS

### 3.1 Histological features

Because site‐dependent differences were more clearly seen between the positive and negative control nodes when compared between the proximal and distal nodes in patients with lower‐lobe cancer, observations of the control nodes will be shown first.

#### 3.1.1 Positive control node

**FIGURE 1:**
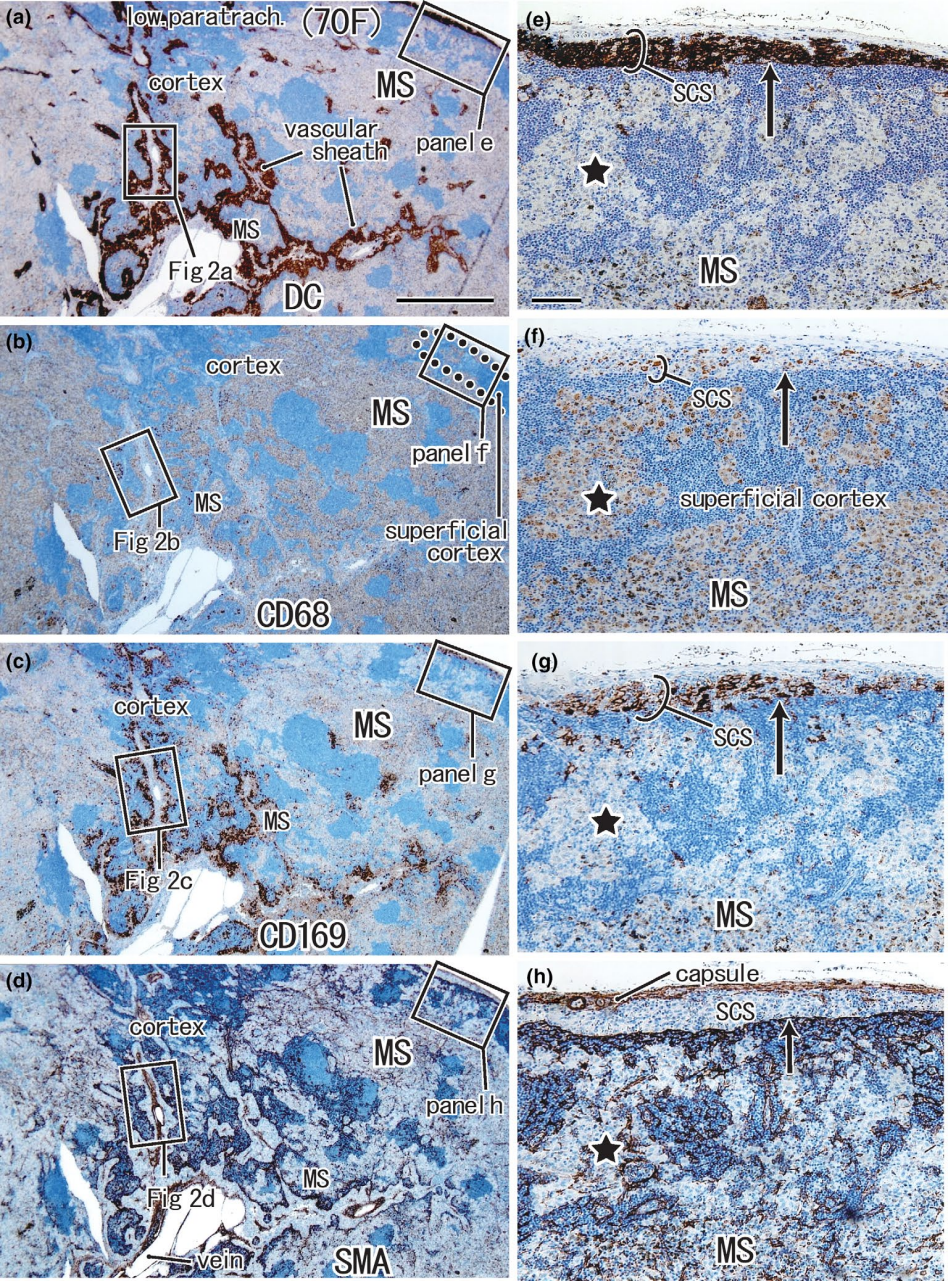
Positive control node (lower paratracheal node) from a 70‐year‐old female patient with upper‐lobe lung cancer. Panels (a)–(d) display adjacent or near sections. Panels (e)–(h) show higher magnification views of the squares in panels (a)–(d), respectively. Immunohistochemistry for DCsign (panels a and e), CD68 (panels b and f), CD169 (panels c and g), and smooth muscle actin or SMA (panels d and h). DCsign‐positive cells are observed in the subcapsular sinus (SCS) and along the vascular sheath (panels a and e). A higher magnification of the vascular sheath (squares with figure number in panels a–d) is shown in Figure 2. The subcapsular sinus (SCS) is filled with DCsign‐positive cells and CD169‐positive cells, but it contains few CD68‐positive macrophages (long arrow in panels e–g). The nodal capsule and endothelium of the sinuses express SMA (panel h). SMA‐positive endothelia are also observed in lymphocyte clusters (i.e., the cortex). The medullary sinus (MS) inserts into the superficial cortex (star in panels e–g) and contains abundant CD68‐positive and CD169‐negative macrophages. Panels (a)–(d) or panels (e)–(h) were prepared at the same magnification (scale bars, 1 mm in panel a; 0.1 mm in panel e).

**FIGURE 2:**
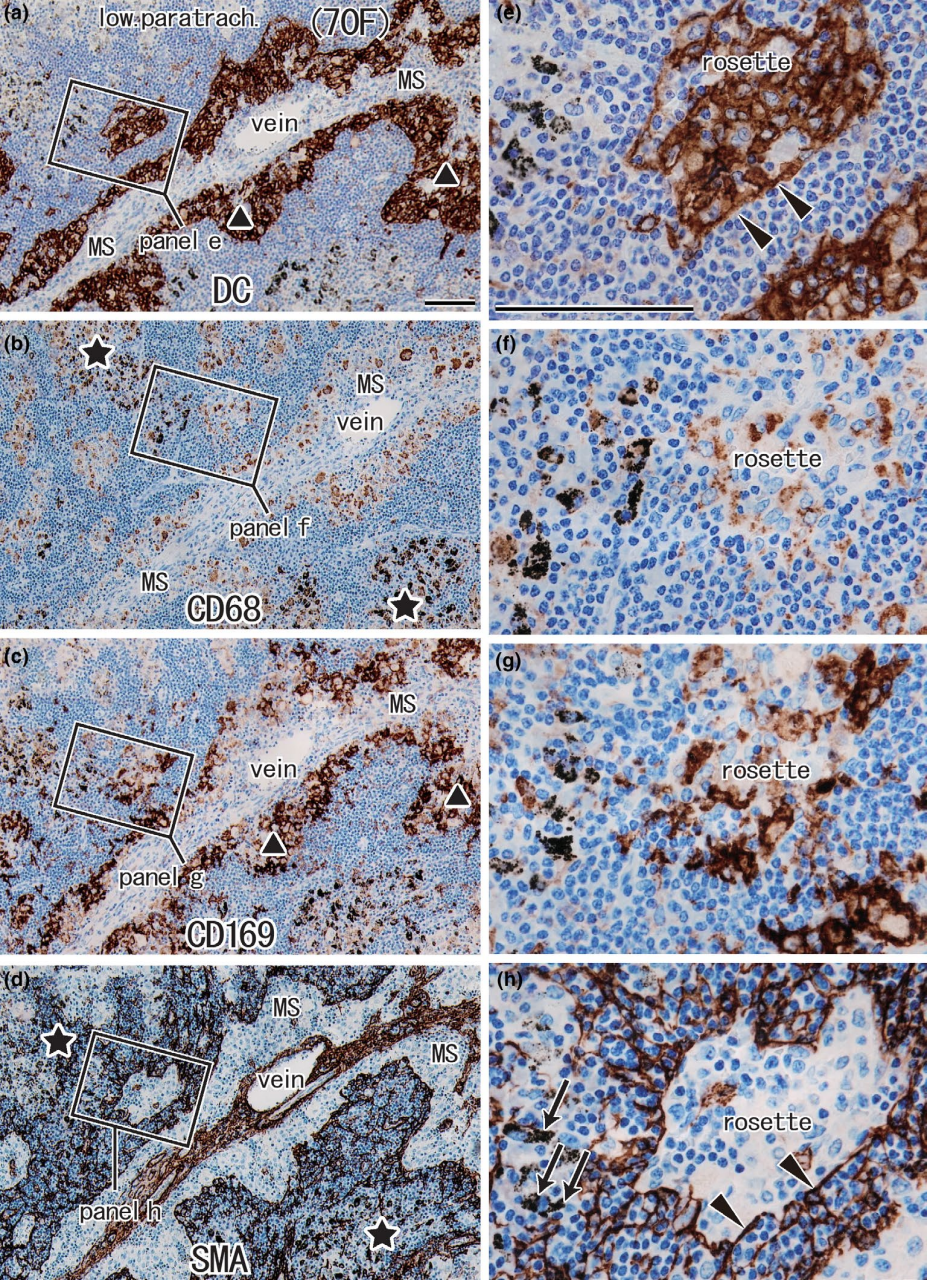
Higher magnification views of DCsign‐positive cells and macrophage clusters in the medullary sinus and cortex (same specimen as Figure 1). Panels (a)–(d) correspond to squares in Figure 1(a)–(d), but the up‐down orientation is tilted at a right angle to save space. Panels (e)–(h) display higher magnification views of squares in panels (a)–(d). Immunohistochemistry for DCsign (panels a and e), CD68 (panels b and f), CD169 (panels c and g), and smooth muscle actin or SMA (panels d and h). DCsign‐positive cell clusters largely overlapped with clusters of CD169‐positive cells in the paracortex near the medullary sinus (MS) (see sites indicated by triangles in panels a and c). A rosette of DCsign‐positive cells is surrounded by DCsign‐positive SMA‐positive endothelium (arrowheads in panels e and h). The rosette did not contain CD68‐positive macrophages but contained CD169‐positive cells. Clusters of anthracotic macrophages contained abundant SMA‐positive fibers (stars in panels b and d), but the latter appeared fragmented (arrows in panel h). Panels (a)–(d) or panels (e)–(h) were prepared at the same magnification (scale bars, 1 mm in panel a; 0.1 mm in panel e).

The MS was consistently filled with CD68‐positive macrophages, whereas DCsign‐positive cells and CD‐169‐positive cells were usually localized in the subcapsular, intermediate, and PCSs (Figures 1 and 2). Thus, DCsign‐positive cells and CD169‐positive cells tended to coexist (Figure 1c vs. Figure 1c), whereas almost all MS macrophages were CD68‐positive CD169‐negative (Figure 1f vs. Figure 1g). The MS issued thin and thick protrusions into the cortex. The thin protrusion extended along the fibrous vascular sheath directed toward the cortex (Figures 1a and 2a). The thick, finger‐like protrusions also extended into the cortex and, when they were great in number in the section, the cortex appeared to be “islands in a sea of CD68‐positive macrophages” (Figure 1b).

Because these protrusions surrounded a small or fragmented cortex, we identified them as the PCS lined by SMA‐positive endothelia (Figures 1h and 2h). Often or sometimes, the endothelia were DCsign‐negative (Figure 1e vs. Figure 1h). The medullary and PCSs were communicated by abundant protrusions from the former. However, this morphology was different from a well‐known laminar architecture of nodes in which a thick cortex surrounds the MS and the belt‐like border area corresponds to the paracortex. At the peripheral end, the PCS was identified as a rosette‐like structure (Figure 2e). The rosette appeared to be a mesh of PCS endothelia, but SMA and DCsign double‐positive epithelium were present only along the margins of the rosette (Figure 2e vs. Figure 2h). CD68‐negative CD169‐positive cells were likely present in the rosette (Figure 2f vs. Figure 2g), but we did not deny a possibility that some DCsign‐positive cells co‐expressed CD169.

#### 3.1.2 Negative control node

**FIGURE 3:**
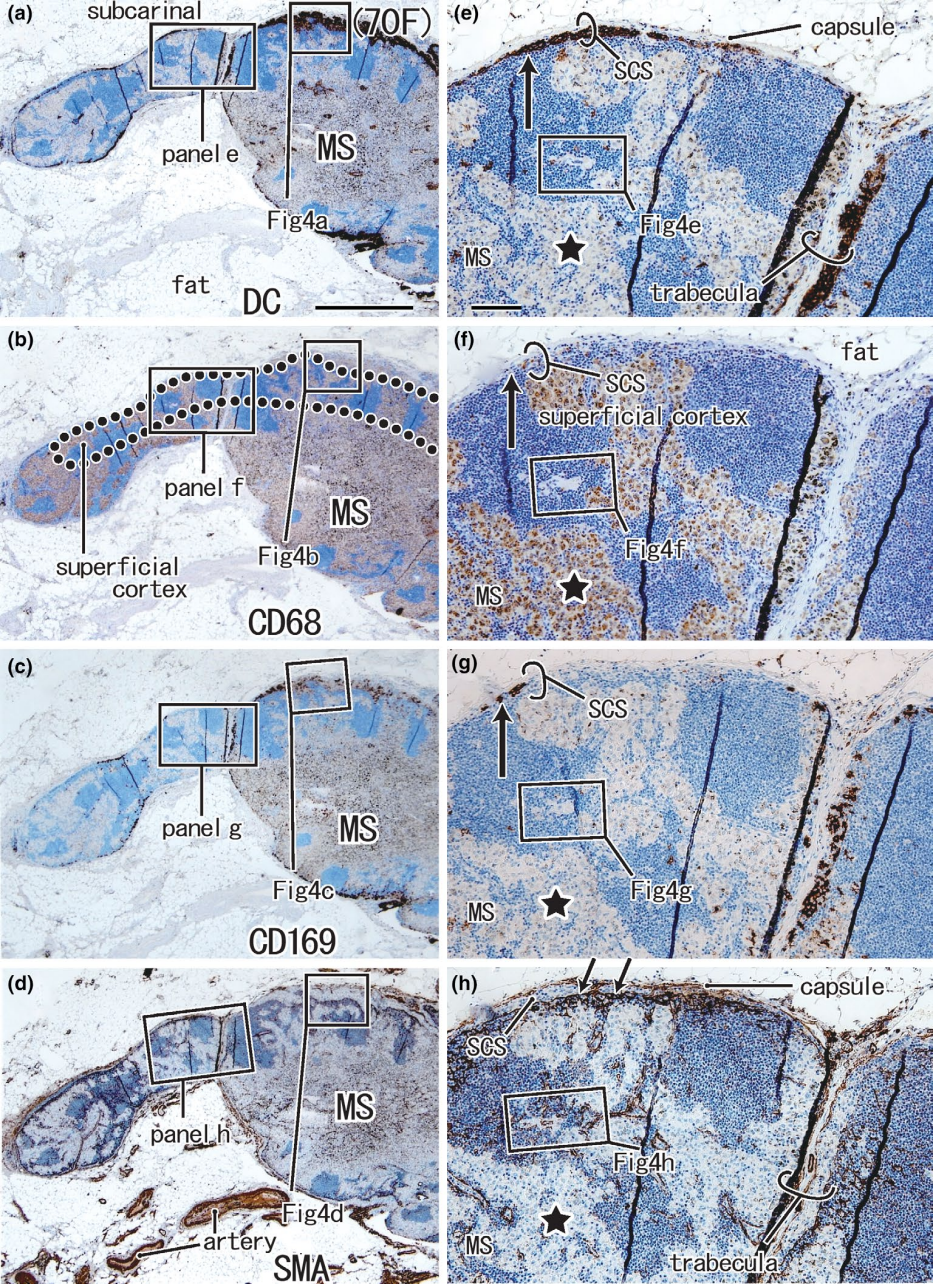
Negative control node (subcarinal node) from a 70‐year‐old female patient with upper‐lobe lung cancer. Panels a‐d display adjacent or near sections. Panels (e)–(h) show higher magnification views of the squares in panels (a)–(d), respectively. Immunohistochemistry for DCsign (panels a and e), CD68 (panels b and f), CD169 (panels c and g), and smooth muscle actin or SMA (panels d and h). DCsign‐positive cells were observed along the nodal surface and trabeculae (panels a and e, respectively). The subcapsular sinus (SCS) is likely to contain DCsign and CD169 double‐positive cells at a site indicated by a long arrow (panels e and g), but CD68‐positive macrophages are absent at the site (long arrow in panel f). The nodal capsule and subcapsular sinus endothelium express SMA (double arrows in panel h). The medullary sinus (MS) contains abundant CD68‐positive CD169‐negative macrophages (stars in panels f and g). Panels (a)–(d) or panels (e)–(h) were prepared at the same magnification (scale bars, 1 mm in panel a; 0.1 mm in panel e).

**FIGURE 4:**
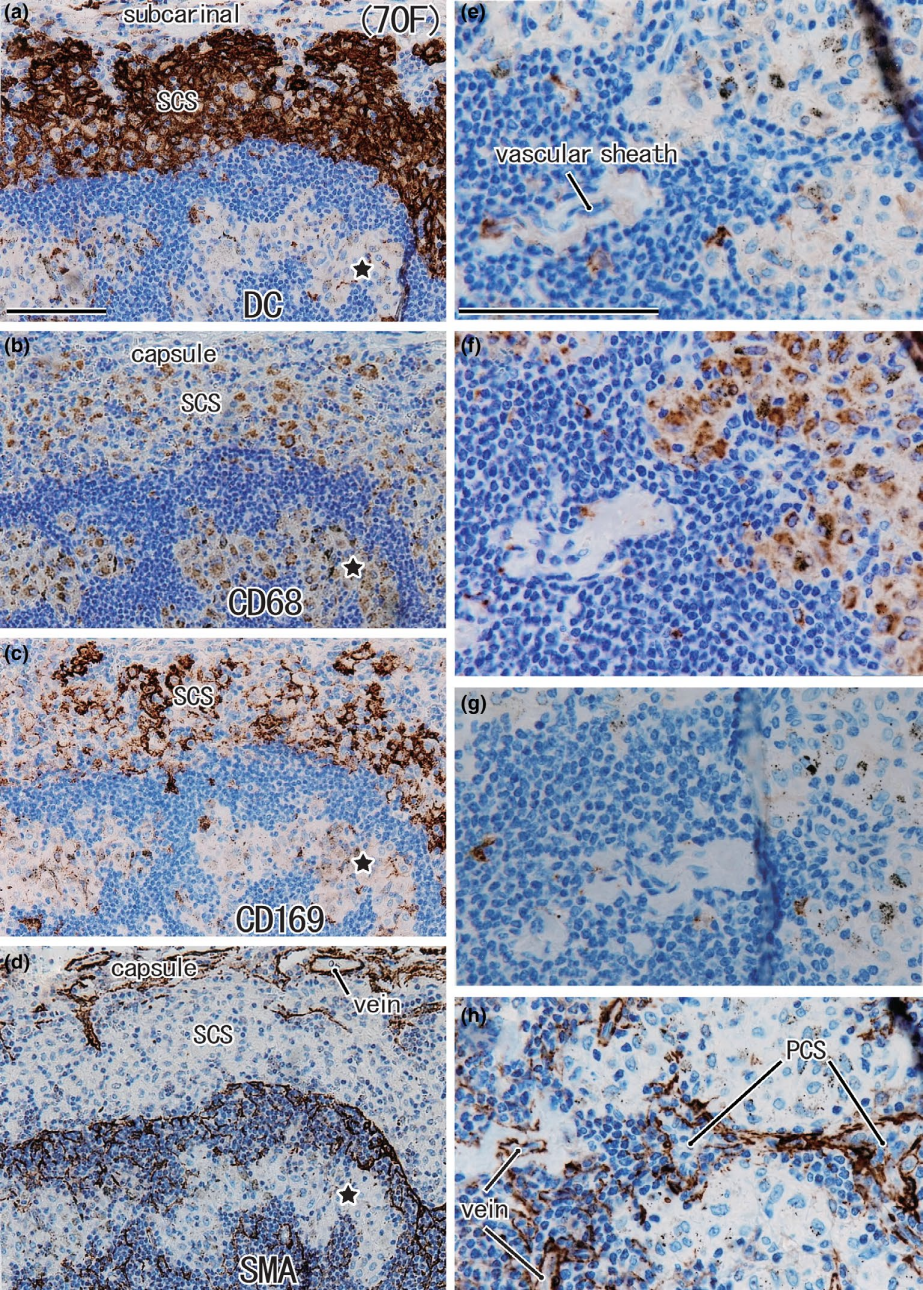
Higher magnification views of DCsign‐positive cells and macrophage clusters in the medullary sinus and cortex (same specimen as Figure 3). Panels (a)–(h) correspond to squares in Figure 1a–h, respectively. Immunohistochemistry for DCsign (panels a and e), CD68 (panels b and f), CD169 (panels c and g), and smooth muscle actin or SMA (panels d and h). DCsign‐positive cell clusters largely overlapped with clusters of CD169‐positive cells in the subcapsular sinus (panels a and c), but abundant CD68‐positive macrophages also exist in the sinus (panel b). The subcapsular sinus is separated from the superficial cortex by SMA‐positive endothelium (panel d). Stars in panels (a)–(d) indicate a protrusion of the medullary sinus that contains no or few DCsign‐positive cells in contrast to abundant CD68‐positive macrophages. Note no or few DCsign‐positive cells and CD169‐positive cells along a vascular sheath and nearby sites (panels e and g). A thin paracortical sinus (PCS) is surrounded by SMA‐positive endothelia (panel h). Panels (a)–(d) or panels (e)–(h) were prepared at the same magnification (scale bars, 0.1 mm in panels a and e).

The complementary distribution was also seen between CD68‐positive macrophages and CD169‐positive cells (Figures 3 and 4). The typical intermediate sinus along a trabecula is included in Figure 3e: it originated from the nodal capsule and inserted into and divided into the superficial cortex. However, relative to the positive control node, the subcapsular and intermediate sinuses tended to contain abundant CD68‐positive macrophages (Figures 3f and 4b). Finger‐like thick protrusions of the MS divided the cortex and made an island‐like appearance (Figure 3e vs. Figure 3f). SMA‐positive endothelium was evident at the border between the SCS and superficial cortex as in the positive control (Figure 3h vs. Figure 1h). However, endothelia of the PCS, surrounding the fragmented cortex, tended not to express SMA reactivity (Figure 3h vs. Figure 1h). Likewise, a vascular sheath sometimes fails to accompany protrusions from the MS (Figure 4e–g).

#### 3.1.3 Proximal and distal nodes

**FIGURE 5:**
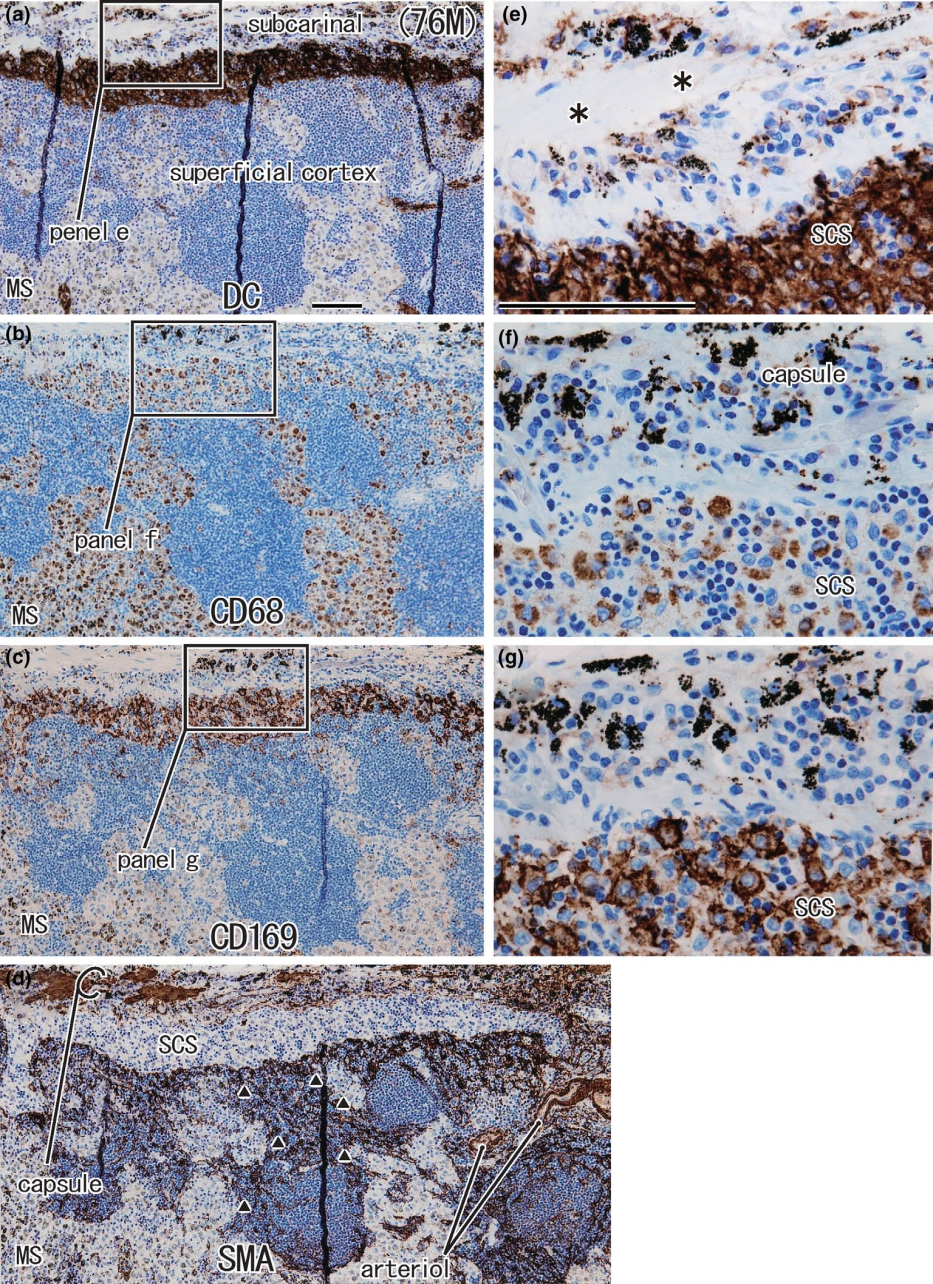
Proximal node (subcarinal node) from a 76‐year‐old male patient with lower‐lobe lung cancer. Panels (a)–(d) display adjacent or near sections. Panels (e)–(g) show higher magnification views of the squares in panels (a)–(c), respectively. Immunohistochemistry for DCsign (panels a, e), CD68 (panels b, f), CD169 (panels c, g), and smooth muscle actin or SMA (panel d). The thick subcapsular sinus (SCS) is filled with DCsign‐positive cells and CD169‐positive cells (panels a, c), but CD68‐positive macrophages are also contained (panel f). Asterisks in panel e indicate an artifact slit during the histological procedure. This is a rare node in which the nodal capsule contains anthracotic macrophages (panel f). The capsule also contains smooth muscles (panel d). The superficial cortex is fragmented by thick protrusions of the medullary sinus (MS) and surrounded by SMA‐positive endothelia (triangles in panel d). Panels (a)–(d) or panels (e)–(g) were prepared at the same magnification (scale bars, 0.1 mm in panels a and e).

**FIGURE 6:**
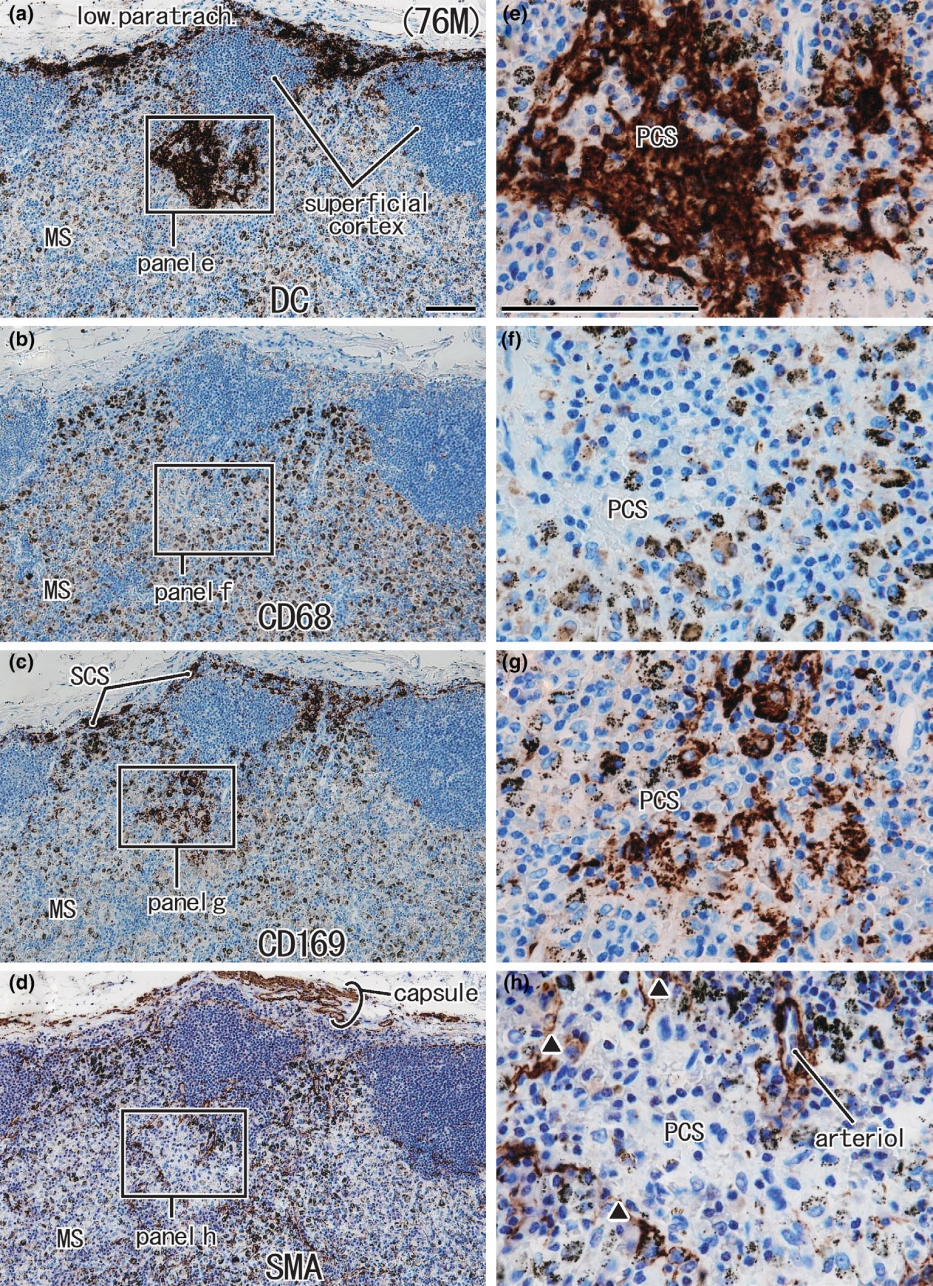
Distal node (lower paratracheal node) from a 76‐year‐old male patient with lower‐lobe lung cancer. Panels (a)–(d) display adjacent or near sections. Panels (e)–(h) show higher magnification views of the squares in panels (a)–(d), respectively. Immunohistochemistry for DCsign (panels a and e), CD68 (panels b and f), CD169 (panels c and g), and smooth muscle actin or SMA (panels d and h). The thin subcapsular sinus (SCS) is filled with DCsign‐positive cells and CD169‐positive cells (panels a and c). The capsule contains smooth muscle (panel d). The superficial cortex is fragmented by thick protrusions of the medullary sinus (MS). A paracortical sinus (PCS) is filled with DCsign‐positive cells (panel e) but SMA‐positive endothelia were not continuous (triangles in panel h). Panels (a)–(d) or panels (e)–(h) were prepared at the same magnification (scale bars, 0.1 mm in panels a and e).

The complementary distribution was seen between CD68‐positive macrophages and CD169‐positive cells, and the latter tended to exist with DCsign‐positive cells (Figures 5 and 6). The island‐like appearance of the cortex was seen both in the proximal and distal nodes (Figures 5a and 6a). Anthracotic macrophages with carbon particles were observed in the MS (Figures 5b,f and 6b,f). Whether the SCS was thick (Figure 5d) or thin (Figure 6c,d) did not depend on the site of nodes, but it appeared to be the result of an individual difference. A difference in SMA‐positive endothelia of the PCS was not evident between proximal and distal nodes (Figures 5d and 6d,h). Overall, there was not a clear difference in general histology between the proximal and distal nodes.

### 3.2 Morphometrical analysis

#### 3.2.1 General feature

A small overlap between clusters of DCsign‐positive cells and CD68‐positive macrophages consistently characterized all the regional nodes examined; it was <10% of the macrophage clusters in more than half of the nodes. Thus, clusters of DCsign‐positive cells and CD68‐positive macrophages usually show a complementary distribution. Because of the common features, there was no statistically significant difference between the proximal and distal nodes (*p* = 0.421; Table 4) or between the positive and negative control nodes (*p* = 0.718). The baseline level of the overlap, defined by the negative control data from the outsider node, was (1) 9.6 ± 7.2% of CD68‐positive cell clusters in the DCsign‐positive cell area (overlap between clusters of DCsign‐positive cells and CD68‐positive macrophages); (2) 6.2 ± 5.4% of CD169‐positive cell clusters in the CD68‐positive area (overlap between CD68‐positive cells and CD169‐positive cells); and (3) 21.2 ± 10.2% of CD169‐positive cell cluster in the DCsign‐positive cell area (overlap between clusters of DCsign‐positive cells and CD169‐positive cells).

#### 3.2.2 Proximal and distal nodes

In 16 patients with lower lung cancer, the total sectional area ranged from 3.5 to 33.9 mm^2^ in the proximal node and from 4.0 to 65.0 mm^2^ in the distal node: the difference was not statistically significant (Table 1). Likewise, the macrophage and CD169‐positive cell areas showed no significant difference between the proximal and distal nodes. Conversely, in the subcapsular cell clusters (Table 3), CD68‐positive macrophages occupied significantly longer SCSs in the distal node (*p* = 0.034). CD169‐positive cells also tended to occupy the longer SCS in the distal node, but the difference was not statistically significant (*p* = 0.458). Notably, in the parameters of overlapping cluster areas (Table 4), the proximal node carried a significantly smaller overlap between clusters of DCsign‐positive cells and CD169‐positive cells than the distal node (*p* = 0.015). In Table 4, we hypothesized that >30% of overlap (or <20%) corresponds to a high (or low) anticancer activity.

**TABLE 3:** Subcapsular clusters of DCsign‐positive cells and macrophages in patients with lower‐lobe cancer.

Age and sex	Site of nodes	Circumferential length of node mm	Subcapsular DCsign/circumference%	Subcapsular CD68‐macrophage/circumference%	Subcapsular CD169‐macrophage/circumference%
56 M	Proximal	28.3	59.0	8.9	31.7
	Distal	38.9	12.5	7.1	13.1
56F	Proximal	13.8	30.1	29.6	22.3
	Distal	15.3	10.5	28.6	9.7
57F	Proximal	16.9	43.7	11.8	39.9
	Distal	8.3	58.3	17.0	42.9
63F	Proximal	13.4	29.1	49.9	34.4
	Distal	30.9	22.3	42.7	21.2
63F	Proximal	26.6	34.1	9.3	35.3
	Distal	6.6	44.4	13.4	29.3
64 M	Proximal	12.5	40.2	21.3	43.3
	Distal	12.3	37.4	20.5	21.3
64F	Proximal	33.7	30.4	11.8	4.8
	Distal	31.9	49.4	11.5	23.6
65 M	Proximal	14.6	18.6	11.0	23.5
	Distal	13.6	15.1	26.3	26.9
66 M	Proximal	17.9	35.7	11.4	25.2
	Distal	15.2	38.9	18.0	33.3
67 M	Proximal	23.3	16.7	12.3	24.2
	Distal	32.5	28.5	7.6	22.1
71F	Proximal	17.5	41.1	20.2	23.5
	Distal	17.6	54.7	26.5	43.9
72 M	Proximal	28.6	39.0	17.1	28.5
	Distal	13.2	47.7	40.7	48.6
73 M	Proximal	22.6	27.9	1.6	0.0
	Distal	17.1	37.7	24.5	31.8
73 M	Proximal	19.9	41.0	11.0	25.7
	Distal	26.7	43.6	23.3	23.9
76 M	Proximal	24.0	38.6	21.6	35.3
	Distal	25.1	34.0	38.7	29.9
78F	Proximal	22.1	30.0	27.0	17.4
	Distal	26.3	31.2	24.0	17.3
Mean	Proximal	20.5	35.7	18.0	26.7
	Distal	20.0	36.4	26.0	29.8
	*p*‐value	0.852	0.869	0.034*	0.458

* *p* < 0.05.

**TABLE 4:** Overlapped clusters and evaluation of anticancer activity in patients with lower‐lobe cancer.

Age and sex	Site of nodes	DCsign‐CD68/CD68 area%^a^	CD169‐CD68/CD68 area%^b^	CD169‐DCsign/DCsign area%^c^	Evaluation of reactivity to cancer^d^
56 M	Proximal	21.5	23.5	36.5	High
	Distal	24.3	24.7	32.7	High
56F	Proximal	19.6	15.4	39.5	High
	Distal	7.6	14.9	47.2	High
57F	Proximal	3.8	5.6	25.7	Moderate
	Distal	16.9	7.6	41.8	High
63F	Proximal	13.3	7.9	42.4	High
	Distal	4.0	4.6	34.6	High
63F	Proximal	4.5	2.8	43.5	High
	Distal	0.5	0.3	41.9	High
64 M	Proximal	9.3	21.9	33.3	High
	Distal	12.8	13.4	14.6	Low
64F	Proximal	3.2	14.2	6.7	Low
	Distal	5.5	3.7	24.6	Moderate
65 M	Proximal	12.1	48.3	11.8	Low
	Distal node	9.1	36.2	8.0	Low
66 M	Proximal	4.5	2.3	29.5	Moderate
	Distal	6.6	5.6	34.0	High
67 M	Proximal	4.9	13.1	35.5	High
	Distal	3.3	2.5	24.2	Moderate
71F	Proximal	6.0	6.8	19.0	Low
	Distal	15.5	10.3	41.9	High
72 M	Proximal	5.5	36.2	23.1	Moderate
	Distal	27.9	26.6	54.6	High
73 M	Proximal	19.3	18.0	23.5	Moderate
	Distal	8.4	46.3	24.2	Moderate
73 M	Proximal	6.9	6.6	21.3	Moderate
	Distal	11.9	7.3	20.2	Moderate
76 M	Proximal	2.7	2.9	34.4	High
	Distal	7.6	35.3	46.8	High
78F	Proximal	5.4	4.0	34.3	High
	Distal	16.0	20.5	27.4	Moderate
Mean	Proximal	8.8	14.9	26.6	Moderate
	Distal	10.9	16.8	36.2	High
	*p*‐value	0.421	0.683	0.015*	

^a^ A proportion of the area overlapped with DCsign‐positive cell cluster in CD68‐positive macrophage areas of the node.

^b^ A proportion of the area overlapped with CD169‐positive cell cluster in CD68‐positive macrophage areas of the node.

^c^ A proportion of the area overlapped with CD169‐positive cell cluster in DCsign‐positive cell cluster of the node.

^d^ Evaluation of reactivity: high, CD169‐positive cell cluster in DCsign‐positive cell cluster >30%; moderate, 20%–29%; low, <20% (See the first paragraph of the Section 4).

* *p* < 0.05.

#### 3.2.3 Control nodes

In the control group of nodes from 10 patients with upper‐lobe cancer, the total sectional area ranged from 10.9 to 24.4 mm^2^ in the positive control node and from 4.0 to 65.0 mm^2^ in the negative control node. There was no statistically significant difference between them (partly shown in Table 1). A limited exception was found in a significantly large overlap between clusters of DCsign‐positive cells and CD169‐positive cells in the positive control node (*p* = 0.006). DCsign‐positive cells and CD169‐positive cells tended to carry longer subcapsular clusters in the positive control node (*p* = 0.083 and 0.05, respectively). Overall, the morphometrical difference was more evident between the positive and negative control nodes relative to a comparison between the proximal and distal nodes.

#### 3.2.4 Other parameters of patients and cancers

The smoking index was significantly correlated with the area of overlap between CD68‐ and CD169‐positive cells (*p* = 0.051) and was higher in men than in women (*p* = 0.04). Notably, irrespective of the cancer pathology and whether the primary lesion occupied the upper or lower lobe, the tumor size, including the marginal invasion around the primary lesion, was significantly correlated with the amount of subcapsular DCsign‐positive cells and CD169‐positive cells (*p* = 0.003, 0.043). In addition, when the pathology was adenocarcinoma, CD68‐positive macrophages showed a strong tendency to occupy the larger SCS of any node (*p* = 0.049). In contrast, in patients with squamous cell carcinomas, CD169‐positive cell proportion showed a strong tendency to be high in all nodes (*p* = 0.042). However, the latter result did not indicate the fact that the node contained a larger number of CD169‐positive cells because the paracortical clusters were much larger than the subcapsular clusters.

## 4 DISCUSSION

The present study demonstrated a significantly larger overlap between clusters of DCsign‐positive cells and CD169‐positive cells in the distal node relative to the proximal node. A DCsign‐ CD169‐double positive cell, which was reported by Yamada et al. (2023), was likely to be contained in the overlapped area, but we considered it was limited in number. Therefore, we hypothesized that, in the distal nodes, the larger overlap corresponded to a stronger cross‐presentation of antigens between DCs and CD169‐positive cells. The cross‐presentation induces further DC maturation (Grabowska et al., 2018). Conversely, the proximal node appeared to have already been suppressed by cancer.

Sinus endothelia were often clearly identified in the distal node, especially in the paracortex, in relation to the proximal node, which might be due to the increased numbers of DCsign‐positive cells and CD169‐positive cells. However, the related information presented in the references is relatively simple: lymph sinus endothelial proliferate and reconstruct before homing cancer cells (Commerford et al., 2018; Lei et al., 2022; Wei et al., 2021). The CD169‐expression level was significantly downregulated in patients with lung cancer, according to gene expression profiling (Zhang et al., 2021). At the baseline or minimum level, the overlapped area was 21.2% in lung cancer patients (data from the negative control in this study) and 44.7% in gastric cancer patients (data from non‐sentinel nodes in Sonoda et al., 2025). Therefore, the level of downregulation of nodes differed between lung and gastric cancers. A large number of alveolar macrophages seemed to decrease the anti‐cancer reaction.

We evaluated the anticancer activity based on the co‐existence between DCsign‐positive cells and CD169‐positive cells (final column of Table 4). The estimated reaction showed a remarkable individual difference. Rather than proximal nodes being suppressed by cancer antigens coming earlier (evaluated “low”), distal nodes seemed to play an active role against cancer even in patients with early cancer (evaluated “high”). We further classified the 16 patients into 3 categories: the proximal node showed higher activity than the distal node (5 patients), 2) both proximal and distal nodes showed high activity (5 patients), and 3) the distal node showed higher activity than the proximal node (6 patients). Therefore, individual differences were also evident, although the suppression or degeneration had started in the proximal node.

It is not widely known that most human SCS macrophages express CD169, as this has only recently been reported in colic and gastric regional nodes (Yamada et al., 2023; Sonoda et al., 2025). Indeed, some research groups have made a negative comment on the active role of CD169‐positive macrophages in cancer immunity: (1) when CD169‐positive macrophages are associated with tumors, the macrophages promote metastasis (Gunnarsdottir et al., 2023); and (2) SCS CD169‐positive macrophages in lymph nodes provide anchorage of metastatic melanoma cells (Singh & Choi, 2019). However, the former study did not consider the colocalization between CD169‐positive cells and DCsign‐positive cells, while the latter study did not observe macrophages in the lymph nodes, rather than tumor‐associated macrophages.

Subcapsular macrophages are considered immature cells “waiting” for antigens from afferent lymph vessels (Engering et al., 2004; Yamada et al., 2023). Notably, the size of the tumor, including the marginal invasion around the primary lesion, was found to have a significant correlation with the amount of monocyte migration, which was strongly suggested by larger subcapsular clusters of DCsign‐positive cells and CD169‐positive cells (*p* = 0.003 and 0.043, respectively). The amount of dispersed cancer antigens seemed to depend on tumor size.

Aoki et al. (2023) emphasized a complementary distribution between DCsign‐positive cells and CD68‐positive macrophages in the lung regional node. The overlapping area was <10% of the entire area of macrophage clusters in more than half of the nodes analyzed in the present study and a previous study by Aoki et al. (2023). This was quite different from gastric regional nodes, in which a small overlap of <10% is unlikely (14.4% at minimum; Sonoda et al., 2025). Thoracic nodes generally contain anthracotic alveolar macrophages in both the MS and cortex (Jin et al., 2022). Alveolar macrophages are self‐renewing populations that arise from fetal progenitors and require minimal input from adult monocytes in a healthy environment (reviewed by Misharin et al., 2017). Indeed, CD68‐positive CD169‐negative macrophages occupied significantly longer SCSs in the distal node, but this might be a result of the delayed “mobilization” of alveolar macrophages by local inflammation associated with cancer.

At the beginning of this study, we hypothesized that the lower paratracheal node is a candidate proximal node for upper‐lobe cancers. Beyond our expectations, it was a good positive control for the present study design because of the greatest overlapping distribution of DCsign‐positive cells and CD169‐positive cells. However, the lower paratracheal node is likely to correspond to the end node because of collateral vessels draining directly to the venous angle (Murakami et al., 2002). Advanced upper‐lobe cancer always makes a metastasis in the paratracheal node, but the hilar node is sometimes free from metastasis because of collateral routes (Maniwa et al., 2018). The pulmonary hilar node, which is characterized by a small cortex (Murakami et al., 2002; Taniguchi et al., 2004), is also likely to be near the upper‐lobe lesions. Further studies including the pulmonary hilar nodes are necessary.

### 4.1 Conclusive remark

In the lung regional nodes of early cancer patients, the distal node was characterized by a large overlapping area between clusters of DCsign‐positive cells and CD169‐positive cells. Conversely, the proximal node seemed to have already been suppressed by the cancer. We believe that the present methodology provides new insights into this field of research.

## 5 STUDY LIMITATIONS

At the beginning of this study, we noted the specific position of the subcarinal node along the lymph flow from the lung (see Section 1) used as a negative control. However, there is an essential limitation: in patients with “upper” lobe cancer, the subcarinal node is difficult to obtain because of the regulation of surgical procedures by clinical academies in Japan. A clinical study is necessary to determine the reliability of our hypothesized definition of high or low activity of the cross‐presentation of cancer antigens (overlap of DCsign‐positive cells and CD169‐positive cells >30% or <20%).

## AUTHOR CONTRIBUTIONS

G.M. designed the study; M.A., G.K., A.H‐T., T.N., and U.K. collected anatomical specimens; M.A. analyzed and visualized anatomical data; M.A. drafted the manuscript; G.M. and U.K. revised critically. All authors read and approved the final manuscript.

## CONFLICT OF INTEREST STATEMENT

The authors declare no conflicts of interest in association with the present study.

## Data Availability

The data that support the findings of this study are available on request from the corresponding author. The data are not publicly available due to privacy or ethical restrictions.
